# Mobile Phone Apps for Preventing Cancer Through Educational and Behavioral Interventions: State of the Art and Remaining Challenges

**DOI:** 10.2196/mhealth.5361

**Published:** 2016-05-30

**Authors:** Steven Coughlin, Herpreet Thind, Benyuan Liu, Nicole Champagne, Molly Jacobs, Rachael I Massey

**Affiliations:** ^1^ University of Massachusetts Lowell Department of Community Health and Sustainability Lowell, MA United States; ^2^ University of Massachusetts Lowell Department of Computer Science Lowell, MA United States; ^3^ Lowell Center for Sustainable Production Lowell, MA United States; ^4^ Toxics Use Reduction Institute Lowell, MA United States

**Keywords:** mobile phone apps, cancer, early detection of cancer, diet, environmental carcinogens, health literacy, nutrition, obesity, prevention, randomized controlled trials, screening, smoking, sun safety, weight loss

## Abstract

**Background:**

Rapid developments in technology have encouraged the use of mobile phones in smoking cessation, promoting healthy diet, nutrition, and physical activity, sun safety, and cancer screening. Although many apps relating to the prevention of cancer and other chronic diseases are available from major mobile phone platforms, relatively few have been tested in research studies to determine their efficacy.

**Objective:**

In this paper, we discuss issues related to the development and testing of new apps for preventing cancer through smoking cessation, sun safety, and other healthy behaviors, including key methodologic issues and outstanding challenges.

**Methods:**

An exploratory literature review was conducted using bibliographic searches in PubMed and CINAHL with relevant search terms (eg, smartphones, smoking cessation, cancer prevention, cancer screening, and carcinogens) to identify papers published in English through October 2015.

**Results:**

Only 4 randomized controlled trials of the use of mobile phone apps for smoking cessation and 2 trials of apps for sun safety were identified, indicating that it is premature to conduct a systematic search and meta-analysis of the published literature on this topic.

**Conclusions:**

Future studies should utilize randomized controlled trial research designs, larger sample sizes, and longer study periods to better establish the cancer prevention and control capabilities of mobile phone apps. In developing new and refined apps for cancer prevention and control, both health literacy and eHealth literacy should be taken into account. There is a need for culturally appropriate, tailored health messages to increase knowledge and awareness of health behaviors such as smoking cessation, cancer screening, and sun safety. Mobile phone apps are likely to be a useful and low-cost intervention for preventing cancer through behavioral changes.

## Introduction

There has been increasing interest in the use of mobile phone apps to promote smoking cessation, healthy eating, physical activity, and other behaviors associated with reduced risk of cancer morbidity and mortality [1-6]. Mobile phone apps have lowered costs, reduced the burden to participants, and overcome some limitations of traditional in-person behavioral weight loss programs [4-6]. Established interventions for smoking cessation and weight loss are resource-intensive, a factor that poses barriers for full participation and widespread dissemination. Mobile phone apps provide a useful and low-cost way to disseminate cancer prevention and control information to the general population and to particular at-risk populations [4].

Rapid technological advances have led to the emergence of smartphones that combine the voice and text messaging functions of mobile phones with powerful computing technologies that can support third-party apps, access to the Internet, and wireless connectivity with other devices [7]. The boom in mobile health (mHealth) has been made possible by the high penetration of Internet access, lower-cost access to broadband Internet, improvement of morbid supporting services, and increased use of smartphones [8]. About 58% of adults in the United States owned a smartphone in 2013 and the percentage is projected to surpass 90% by 2020 [8,9]. About 64% of African Americans, 60% of Hispanics, and 53% of Caucasians in the United States owned a smartphone [10]. In addition to seeking health information, people use health apps to monitor their own health conditions and to manage their health [8]. For example, 38% of health app users use an app to track their exercise [11].

A mobile app is a computer program designed to run on smartphones or other mobile devices. All major smartphone platforms provide third-party developers with app programming interfaces that can be used to build special purpose apps referred to as native apps [7]. Smartphone apps can have a variety of features including visually engaging design, video and audio capabilities, unrestricted text capabilities, access without cellular or Internet connection, optimized smartphone screen size, content sharing via social media, and tracking progress anywhere and anytime [12]. In April 2012, there were an estimated 13,600 consumer health apps for the iPhone. The rapid increase in mobile apps has led to the proliferation of blogs, magazines, and dedicated online app-discovery services [13].

A variety of apps relating to cancer prevention, smoking cessation, diet, nutrition, and weight control are available from major smartphone platforms such as iPhone, Android, Nokia, and BlackBerry. Common behavioral change techniques include providing feedback, goal-setting, self-monitoring, and planning social support and change [14]. However, relatively few have been tested in randomized controlled trials to determine their efficacy in promoting health [7,15]. In addition, few of these apps are based on theories of health behavior change, most do not include evidence-based features, such as reinforcement, and existing apps often do not provide evidence-based recommendations for cancer prevention [1,7,15,16]. Furthermore, few studies have examined the general cognitive motivators that prompt people’s use of health apps [8].

In this viewpoint, we discuss issues related to the development and testing of new apps for preventing cancer through smoking cessation, healthy diet and nutrition, physical activity, weight management, cancer screening, and sun safety, including key methodologic issues and outstanding challenges related to methodology, design issues, and regulatory issues. In an exploratory literature review intended to inform our commentary, we review published studies on the acceptability and effectiveness of mobile phone apps designed to promote behaviors that reduce risk of cancer [17,18].

Of particular interest were randomized control trials of the effectiveness of mobile phone apps to promote healthy behaviors such as smoking cessation and sun safety. Although mobile health apps related to telemedicine, cancer diagnosis and treatment, and oncology clinical trials also hold promise for cancer prevention and control [19-21], the focus of this review is on mobile phone apps for promoting healthy behaviors and cancer risk reduction through educational and behavioral interventions. As there have been recent reviews of the use of mobile phone apps to promote healthy diet, nutrition, and physical activity [5,6], we narrowed the focus of our exploratory literature review to randomized controlled trials of the use of mobile phone apps for smoking cessation, cancer prevention, cancer screening, or avoiding carcinogens.

## Methods

We conducted bibliographic searches in PubMed and CINAHL with relevant search terms: (smartphones) and ((smoking cessation) or (cancer prevention) or (cancer screening) or (carcinogens)). Papers published in English through October 2015 were identified using relevant MeSH search terms and Boolean algebra commands. The searches were not limited to words appearing in the title of a paper. Studies that did not have a randomized controlled or pre-post test design were excluded along with those that focused on patients with cancer or other chronic diseases (ie, mobile phone apps for disease management). Randomized controlled trials are the most rigorous study design and the standard for most of the cancer prevention and control interventions (eg, cancer screening). Information obtained from bibliographic searches (title and topic of paper, information in abstract, geographic locality of a study, and key words) was used to determine whether to retain each paper identified in this way. We also looked for relevant papers published in *JMIR mHealth and uHealth* and *JMIR Cancer*. In addition, we identified reports included in Cochrane reviews [22] and reviewed the references of published review articles. More general searches were conducted that focused on eHealth literacy and methodologic issues in the development of mobile health apps.

## Results

**Figure 1:**
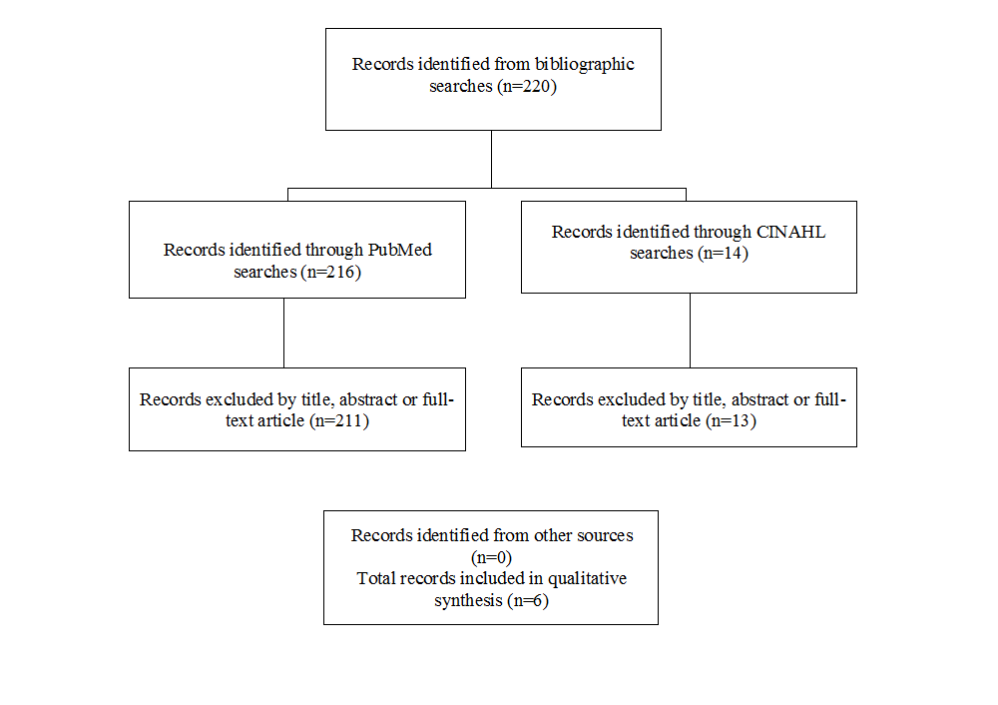
Summary of search and exclusion process for identified randomized controlled trials.

A total of 220 papers were identified in the bibliographic searches (Figure 1). By screening abstracts or full-text articles, 4 randomized controlled trials of the use of mobile phone apps for smoking cessation were identified (Table 1). Valdivieso-Lopez et al [23] designed a cluster randomized controlled trial of a mobile phone app for smoking cessation in primary care centers in Catalonia, Spain. The efficacy of the mobile phone app combined with clinical practice guidelines for smoking cessation will be compared with clinical practice guidelines alone. The participants in the 6-month intervention trial will be smokers who have 10 or more cigarettes per day, aged 18-30 years, who are motivated to quit smoking. The outcome measure will be abstinence at 12 months confirmed by exhaled air carbon monoxide concentration. The app is being designed as a serious game that offers users the opportunity to develop skills and strategies for smoking cessation while trying to achieve the game’s objectives. Results from the trial have not been published.

**Table 1:** Randomized controlled trials of mobile phone apps for promoting smoking cessation.

Study	Sample	Design	Results	Other information
Valdivieso-Lopez et al (2012)	Smokers of 10 or more cigarettes per day, aged 18-30 years who are motivated to quit smoking, seen in primary care centers in Catalonia, Spain	Cluster randomized controlled trial of a mobile phone app for smoking cessation combined with clinical practice guidelines, compared with clinical practice guidelines alone. The outcome measure will be abstinence at 12 months confirmed by exhaled air carbon monoxide concentration	Not yet available	The app is being designed as a serious game which offers users the opportunity to develop skills and strategies for smoking cessation while trying to achieve the game’s objectives
Buller et al. (2014)	102 US smokers aged 18 to 30 years	Randomized controlled trial of a mobile app compared with text messaging (12-week pretest-posttest trial). Self-reported usability of the mobile phone app and quitting behavior (quit attempts, point prevalence, 30-day point prevalence, and continued abstinence) were assessed in posttests	A sizeable percentage of smokers reported being abstinent at 12-weeks (66% of smokers who completed the intervention trial, 44% of all smokers). Those in the text messaging group were more likely to be abstinent than those in the mobile phone app group (*P* <.05)	
Bricker et al 2014	196 adults who smoked at least five cigarettes per day for at least past 12 months, and were motivated to quit and interested to learn skills to quit smoking	Double-blind, randomized controlled trial of the effectiveness of a mobile phone delivered app (QuitGuide) versus the National Cancer Institute’s SmartQuit app for smoking cessation. The outcome measure was self-reported 30-day point prevalence of abstinence (ie, no smoking in the last 30 days).	The overall quit rates were 13% in SmartQuit versus 8% in QuitGuide (odds ratio=2.7, 95% confidence interval 0.8-10.3)	
Baskerville et al. 2015	1354 smokers in Canada, aged 19-29 years	6-month, randomized controlled trial of a mobile phone app (Crush the Crave) versus an evidence-based self-help guide. The primary outcome will be self-reported, 30-day point prevalence of abstinence	Not yet available	

Buller et al [24] conducted a randomized controlled trial of a mobile app compared with text messaging to support smoking cessation. A total of 102 smokers aged 18 to 30 years participated in the 12-week, pretest-posttest trial. Self-reported usability of the mobile phone app and quitting behavior (quit attempts, point prevalence of abstinence, 30-day point prevalence, and continued abstinence) were assessed in posttests. A sizeable percentage of smokers reported being abstinent at 12-weeks (66% of smokers who completed the intervention trial, 44% of all smokers). Those in the text messaging group were more likely to be abstinent than those in the mobile phone app group (*P* <.05).

Bricker et al [8] conducted a double-blind, randomized controlled trial to examine the efficacy of a mobile phone-delivered app (QuitGuide) based upon the acceptance and commitment therapy (ACT) model of behavioral change, compared with the National Cancer Institute’s SmartQuit app for smoking cessation. The latter is based on US Clinical Practice Guidelines. A total of 196 adults, who smoked at least 5 cigarettes per day for at least past 12 months and were motivated to quit and interested to learn skills to quit smoking, participated in the trial. The outcome measure was 30-day point prevalence abstinence (ie, no smoking in the past 30 days). The overall quit rates were 13% in SmartQuit versus 8% in QuitGuide (odds ratio=2.7, 95% confidence interval 0.8-10.3).

Baskerville et al [25] is conducting a 6-month, randomized controlled trial of the efficacy of a mobile phone app (Crush the Crave) compared with an evidence-based self-help guide. A total of 1354 smokers in Canada, aged 19-29 years, will be randomized to the trial. The primary outcome is self-reported, 30-day point prevalence of abstinence. Results from the trial have not yet been reported.

Only two trials of the use of mobile phone apps for promoting sun safety were identified [9,26]. Our bibliographic search did not identify any published randomized controlled trials of the use of mobile phone apps to promote breast or colorectal cancer screening. In addition, there were no published studies regarding the use of apps to help people avoid carcinogenic exposures in work and home environments.

## Discussion

Bender et al [7] examined the purpose and content of cancer-related mobile phone apps available for use by the general public and the evidence for their utility and effectiveness. They systematically reviewed the official stores for the four major mobile phone platforms (iPhone, Android, Nokia, and BlackBerry). Apps were included in their review if they focused on cancer and were available for public use. In addition, they systematically reviewed the literature using MEDLINE, Embase, and the Cochrane Library to identify evaluations of cancer-related mobile phone apps. A total of 295 apps from mobile phone app stores met their inclusion criteria. The reported app purpose was to raise awareness about cancer (32.2%, 95 of 295), to provide educational information about cancer (26.4%, 78 of 295), assist in early detection (11.5%, 34 of 295), and cancer prevention (2.0%, 6 of 295). Their review of the health literature identified 594 papers, but none was deemed eligible as they did not report an evaluation of a cancer-focused mobile phone app [7]. In addition, 17 apps focused on the early detection of breast cancer. Many of the apps promoted a charitable organization or supported fund-raising efforts. The authors noted several concerns including the lack of evidence of app effectiveness or description of the procedures or data sources (eg, evidence, theory) and discrepancies between information generated on mobile phone apps and evidence-based guidelines [7]. Mobasheri et al [15] reviewed major app stores (Apple iTunes, Google Play, BlackBerry World, and Windows Phone) using breast symptoms and diseases. A total of 185 breast apps were identified, of which 139 (75.1%) focused on breast cancer. Most of the apps were educational (*n* =94) or self-assessment tools (*n* =30). Few of the apps were evidence-based (14.2%) or involved medical professionals in their development (12.8%). Potential patient safety concerns were identified in 29 (15.7%) apps [15]. In March 2014, Bricker et al [12] identified 546 smoking cessation apps in the Apple Store and Google Play that were downloaded to mobile phones an estimated 3.2 million times in the United States and 20 million times worldwide.

The number of randomized controlled trials of the efficacy of mobile phone apps in smoking cessation and sun safety is still modest [9,26]. Differences in study design (eg, choice of a comparison group, outcome measures, and sample size) and mobile phone app functionalities also increase the difficulty of drawing firm conclusions about the effectiveness of apps in promoting behaviors associated with reduced cancer risk. Nevertheless, mobile phone apps can be efficacious in promoting smoking cessation and are likely to be a useful and low-cost intervention for smoking cessation in the general population.

### Design Issues

Mobile apps, such as those used for cancer prevention and control, are developed using emulators in specialized development environments. The development of apps must take into account the wide variety of screen sizes, hardware specifications, and configurations of mobile devices, as well as the need to run on a battery. In order to have an understandable and user-friendly interface, mobile user interface design takes into account display screens and input. The interface of users with their device includes both hardware and software components. Constraints considered by mobile user interface design include the limited attention of users and the device’s screen size in relation to a user’s hand.

Guidelines for developing mobile apps are available from academic and industry sources [27-29]. To help ensure the development of a successful app, guidelines for the development of mobile apps highlight the need for an initial analysis of requirements (understand the basic requirements for a proposed app), technology and strategic planning (find the best technology based on the requirements and to strategically plan a project), design and architecture (identify a suitable design, architecture, and interface for the mobile app), development and coding (begin coding modules while keeping the design in mind), testing and approval (test the app on a live server to identify and fix any coding bugs), and implementation [28].

Once developed, prototype apps are subjected to heuristic evaluation and field-testing. Evidence-based heuristics have been developed for evaluating the demands that mobile apps make on users, in terms of health literacy and usability, that is, the extent to which the app is practical and convenient for users [30]. The evaluations identify ways the design of the app could be helpful or detrimental to users with limited eHealth or computer literacy [30]. Heuristics recommended by Monkman et al [30] include:

1. Immediately inform users of purpose and engage users (identify the purpose and audience on the home screen)

2. Use complementary interaction methods (make use of alternative inputs such as touch screen and voice commands) and outputs (eg, audio recordings, videos, and graphics)

3. Leverage interactivity (offer interactive tools such as glossaries, tutorials, and quizzes to engage users with the information and to provide performance feedback)

4. Provide accurate, colloquial, comprehensive, succinct content (written information should be brief, relevant, and in users’ vernacular)

5. Provide tailored, flexible, and layered content (prioritize information according to importance, provide succinct summaries, allow users to access more detailed information, offer content in multiple languages)

6. Use visuals to complement text but avoid tables (visuals such as pictures, videos may enhance written information)

7. Use simplistic consistent navigation (keep users oriented, use linear navigation to facilitate forward and backward movement, use large buttons and clearly labeled links, and provide a search engine)

8. Use simplistic, consistent displays (avoid on screen complexity and the need for scrolling by limiting information on each page or screen).

Simplicity and ease of use are often desirable. For example, if health apps require users to complete multiple steps in order to access information (eg, requiring them to provide numerous details about their meals in order to obtain information about their daily caloric intake), the complex and repetitive process requires users to expend a great deal of their time and mental energy, which can negatively impact their willingness to use the app [14]. It is helpful to complement text information with visuals and to engage users by offering interactive learning tools and resources. To improve readability, low contrast or distracting colors (eg, shadows) should be avoided in an app.

Depending on the app that is being evaluated, it may also be helpful to: (1) provide clear and comprehensive communication of risks (describe risks in ways that users will understand and avoid logarithmic scales); (2) provide clear depiction of monitoring data (facilitate pattern recognition and emphasize values outside of acceptable range); and (3) allow users to adjust the display size using familiar input (eg, pinch to zoom, use appropriately sized interface elements, and limit the amount of information displayed) [30]. Heuristic evaluations provide rapid, low-cost assessments that can help to improve the usability of an app, before conducting more expensive and time-consuming usability testing with samples of users [30].

### Design Challenges

One challenge for the development of mobile phone apps for cancer prevention is that people may be more likely to use an app for a regular behavior such as healthy eating, physical activity, or weight management than an app that focuses on behaviors such as cancer screening that are only required every 5-10 years or biannually. It may be helpful to address multiple cancer prevention behaviors (eg, healthy diet, nutrition, physical activity, and avoidance of known or suspected carcinogens in home or work environments) in the same app [31]. A further issues is that cancer prevention apps that are less popular in the general population could be useful tools for people at increased risk due to a personal or family history of cancer or if recommended by physician (eg, as an adjunct to a smoking cessation program in primary care).

For mobile apps to be useful for preventing and controlling cancer in diverse populations, they must be suitable for people with varying levels of health literacy, eHealth literacy, computer literacy, and scientific literacy. The Institute of Medicine defined health literacy as “the degree to which individuals have the capacity to obtain, process, and understand basic health information and services needed to make appropriate health decisions” [32]. Health literacy is comprises numerical literacy (numeracy), print literacy, and cultural and conceptual knowledge [32]. On average, people with low health literacy have poorer overall health status, inefficient use of health care services, poor patient-provider communication, and higher risk of premature mortality, compared with people with higher health literacy [32-34]. Although few published studies have examined the health literacy levels of educational information provided via mobile apps, in other media, cancer prevention and control messages are often written at too high a reading level for individuals with marginal literacy skills [35]. Health literacy instruments that have been assessed for validity and feasibility as screening tools in clinical settings and in research include, the Test of Functional Health Literacy in Adults (TOFHLA), the Shortened Test of Functional Health Literacy in Adults (S-TOFHLA), the Rapid Estimate of Adult Literacy in Medicine (REALM), and the Shortened Rapid Estimate of Adult Literacy in Medicine (REALM–R) [36-39]. A computer-based version of TOFHLA has been pilot tested [40].

In contrast to the more general concept of health literacy, eHealth literacy is “the ability to seek, find, understand, and appraise health information from electronic sources and apply this knowledge gained to addressing or solving a health problem” [41]. eHealth literacy comprises both general skills and specific skills. General skills include reading, writing, and numeracy, media literacy, and information literacy (ie, information seeking and understanding). Specific skills include computer literacy (information technology skills), health literacy, and science literacy [42,43]. eHealth literacy involves a mix of health, information, scientific, computer, and Internet literacy [44]. In an electronic world where information and communication technology design enables the delivery of health-related information, being health literate requires an expanded set of skills to engage in health promotion and sustain personal health [41,44]. People with higher education are more likely to download health information to a mobile device [45]. Higher educational attainment and younger age are associated with higher eHealth literacy [46]. Rather than being static, both health literacy and eHealth literacy are influenced by an individual’s health status, motivation, education, and changes in technology [34]. Among people with lower socioeconomic status, inequalities likely exist with respect to use of digital resources and online skills [46]. The digital divide relates both to Internet access and to the gap between people who can effectively use new information tools and those who cannot [4].

The model of ehealth literacy proposed by Norman and Skinner, eHEALS, defines the concept using 6 sub-literacies (traditional, information, media, health, computer, and scientific literacy). Eight statements are included (eg, I know how to find helpful health resources on the Internet, I know how to use the health information I find on the Internet to help me) about an individual’s perception of their eHealth literacy measured on a 5-point Likert scale [41]. The eHealth Literacy Assessment Toolkit (eHLA) developed by Furstrand and Kayser [47] provides tools for assessing a user’s familiarity with computers, confidence in using computers, and health literacy. The eHLA toolkit is based upon the work of previous authors [41,48,49]. There has also been interest in developing computer-based health literacy screening instruments suitable for eHealth apps. However, there is currently a lack of information about the psychometric properties of computer-based health literacy instruments [50].

Little information is available about the general cognitive motivators that prompt people’s use of health apps [14]. People who are more health conscious are more likely to use health apps, partly because apps are often used to avoid unhealthy situations or to manage one’s own health condition (eg, obesity, breast cancer). People with higher levels of health consciousness are more likely to have preventive health behaviors (eg, healthy eating habits, exercise, and avoidance of smoking) and actively seek health information from various sources including the Internet [14,51,52]. The concept of self-efficacy, highlighted in Bandura’s social cognitive theory, has been extended to Internet health information use efficacy [53]. Health app use efficacy refers to a person’s cognitive ability to use health apps in order to access health information [14]. Scales have been proposed to measure people’s health consciousness, health information orientation, and health app use efficacy [14,52].

### Regulatory Issues

In some countries, government agencies have begun to regulate or curate medical apps [54-56]. There is concern about both patient safety and the security and confidentiality of patient data transmitted and stored in mobile medical apps [55]. In 2013, the US Food and Drug Administration (FDA) has released guidance for mobile medical apps that draw a distinction between unregulated apps and mobile medical apps that are subject to overt FDA regulation [57]. Apps that convert a mobile platform such as a smartphone or tablet computers into a medical device are regulated by the FDA [55]. The FDA regulates mobile apps that pose a greater risk to patients if they do not function as intended (eg, apps that perform clinical tests such as blood or urine analysis, apps that display diagnostic images from X-rays and magnetic resonance imaging, and those that remotely display data from bedside monitors). Apps for general health education are mostly unregulated [57]. In the United States, Health Insurance Portability and Accountability Act (HIPAA) regulations require covered entities and their business associated (eg, physicians, hospitals, and health plans) to protect health information that identifies an individuals and that relates to an individual’s physical or mental health or health care services to the individual [58]. Mobile app developers must consider whether the software will be used by a covered entity and whether it will include any protected health information. For example, an app that assists a health care provider with following up patients would need to be designed to allow the provider to comply with HIPPA [58]. In the United Kingdom, the National Health Service established a Health Apps Library which endorses apps that are considered to be relevant to people in Britain, provide trustworthy information, comply with data storage regulations, and do not pose potential risks if they are used improperly [59]. A recent assessment of 79 apps certified as clinically safe and trustworthy by the Health Apps Library found systematic gaps in compliance with data protection principles [60]. None of the 79 apps encrypted personal information stored locally, 66% (23 of 35) of apps sending identifying information over the Internet did not use encryption, and 20% (7 of 35) did not have a privacy policy [29]. The authors noted that app users cannot see into the inner workings of apps or the services they connect to; hence, they must trust developers to comply with privacy regulations and security best practices [29]. Medical information stored on apps should be secured using encryption [61]. Systematic reviews of health and wellness apps available from generic app stores have identified deficiencies in the extent to which data users are documented and appropriate security measures are implemented [62,63].

### Conclusions

Additional cancer prevention and control research is needed to examine the efficacy of mobile phone apps [64]. Future studies should utilize randomized controlled trial research designs and adequate sample sizes to better explore the cancer prevention capabilities of mobile phones. The efficacy and effectiveness of mobile phone apps that are already in routine use—for example, the National Cancer Institute’s QuitPal app [65]—should be examined in well-designed randomized controlled trials. There is a need for culturally tailored health messages to increase awareness of behaviors associated with reduced cancer risk such as smoking cessation and sun safety. Research-tested mobile phone apps are also needed for non-English speakers or for persons with low health literacy.

## Abbreviations

ACT: acceptance and commitment therapy
eHLA: eHealth Literacy Assessment Toolkit
FDA: Food and Drug Administration
HIPAA: Health Insurance Portability and Accountability Act REALM: Rapid Estimate of Adult Literacy in Medicine
REALM–R: the Shortened Rapid Estimate of Adult Literacy in Medicine
S-TOFHLA: Shortened Test of Functional Health Literacy in Adults
TOFHLA: Test of Functional Health Literacy in Adults
